# Insights into Protein–Ligand Interactions: Mechanisms, Models, and Methods

**DOI:** 10.3390/ijms17020144

**Published:** 2016-01-26

**Authors:** Xing Du, Yi Li, Yuan-Ling Xia, Shi-Meng Ai, Jing Liang, Peng Sang, Xing-Lai Ji, Shu-Qun Liu

**Affiliations:** 1 Laboratory for Conservation and Utilization of Bio-Resources, Yunnan University, Kunming 650091, China; duxingok@gmail.com (X.D.); liyi.gerry@gmail.com (Y.L.); xiayl@ynu.edu.cn (Y.-L.X.); aishimann@gmail.com (S.-M.A.); liangjingyn90@gmail.com (J.L.); speng431@gmail.com (P.S.); rich@ynu.edu.cn (X.-L.J.); 2 Department of Applied Mathematics, Yunnan Agricultural University, Kunming 650201, China; 3 Laboratory of Molecular Cardiology, Department of Cardiology, The First Affiliated Hospital of Kunming Medical University, Kunming 650032, China; 4 Key Laboratory for Tumor molecular biology of High Education in Yunnan Province, School of Life Sciences, Yunnan University, Kunming 650091, China

**Keywords:** binding mechanisms, thermodynamics, kinetics, binding driving forces, isothermal titration calorimetry (ITC), surface plasmon resonance (SPR), fluorescence polarization (FP), docking, free energy calculations

## Abstract

Molecular recognition, which is the process of biological macromolecules interacting with each other or various small molecules with a high specificity and affinity to form a specific complex, constitutes the basis of all processes in living organisms. Proteins, an important class of biological macromolecules, realize their functions through binding to themselves or other molecules. A detailed understanding of the protein–ligand interactions is therefore central to understanding biology at the molecular level. Moreover, knowledge of the mechanisms responsible for the protein-ligand recognition and binding will also facilitate the discovery, design, and development of drugs. In the present review, first, the physicochemical mechanisms underlying protein–ligand binding, including the binding kinetics, thermodynamic concepts and relationships, and binding driving forces, are introduced and rationalized. Next, three currently existing protein-ligand binding models—the “lock-and-key”, “induced fit”, and “conformational selection”—are described and their underlying thermodynamic mechanisms are discussed. Finally, the methods available for investigating protein–ligand binding affinity, including experimental and theoretical/computational approaches, are introduced, and their advantages, disadvantages, and challenges are discussed.

## 1. Introduction

Molecular recognition refers to the process in which biological macromolecules interact with each other or with various small molecules through noncovalent interactions to form a specific complex [1]. This process has two important defining characteristics: (i) specificity, which distinguishes the highly specific binding partner from less specific partners; (ii) affinity, which determines that a high concentration of weakly interacting partners cannot replace the effect of a low concentration of the specific partner interacting with high affinity [2]. Of greatest importance is the fact that the molecular recognition is not a process in itself, but an element of a more complex, functionally important mechanism involving the essential elements of life—self-replication, metabolism, and information processing. For example, DNA replication occurring before cell division is accomplished by a series of complicated enzyme-catalyzed reactions relying on the recognition and binding between helicase and DNA double helix (responsible for DNA unzipping), DNA polymerase and single strand DNA (responsible for base insertion and DNA synthesis), and ligase and discontinuous DNA segments (responsible for stitching these segments). Similarly, the highly efficient and specific molecular recognition and binding, which act as a prerequisite for enzyme-catalyzed reactions, play a critical role in running and regulating a metabolic network consisting of thousands of chemical reactions running in parallel [3,4]. Cellular signal cascades proceed through a series of recognition, binding, and dissociation events, *i.e.*, they are initiated by small molecular messenger recognition, transmitted via transmembrane receptor segments, and accomplished by the functional response of a cell involved in a multitude of biomolecule binding phenomena [3,5].

Proteins are a very important class of macromolecules because they play a vast variety of roles in the cell, including structural (cytoskeleton), mechanical (muscle), biochemical (enzymes), and cell signaling (hormones) functions. Essentially, proteins realize their biological functions through their direct physical interaction with other molecules, including proteins and peptides, nucleic acids, membrane, substrates, and small molecule ligands such as oxygen, solvent, and metal. For purpose of clarity, in this review we define the “ligand” as any molecule capable of binding to a protein with a high specificity and affinity. A prerequisite for a deeper understanding of protein functions is to understand thoroughly the mechanisms responsible for the protein–ligand interactions, for which the full description, characterization, and quantification of the energetics that govern/drive the formation of a complex are crucial [6]. In addition, because the aim of the rational drug design is to make use of knowledge of the structural data and protein–ligand binding mechanisms to optimize the process of finding new drugs, an in-depth understanding of the nature of the molecular recognition/interaction is also of great importance in facilitating the discovery, design, and development of drugs [7].

This review includes three parts. The first part aims to elucidate the physicochemical mechanisms that govern the protein–ligand association. After introducing the molecular association-relevant concepts such as binding kinetics, free energy, enthalpy, and entropy, the forces/factors that drive the protein–ligand binding are rationalized, followed by an introduction of the phenomenon of entropy–enthalpy compensation and its influence on binding affinity. In the second part, the presently existing three conceptual models describing and interpreting the protein–ligand binding, *i.e.*, the “lock-and-key”, “induced fit” and “conformational selection”, will be introduced and the underlying driving factors and how they work in these models will be discussed. The third part is dedicated to experimental and theoretical methods applied to assess protein–ligand binding affinity. The experimental methods mainly focus on isothermal titration calorimetry (ITC) [6,8], surface plasmon resonance (SPR) [9,10], and fluorescence (de)polarization (FP) [11,12] due to their capacity to provide information both about the binding kinetics and thermodynamics. The computational methods introduced include the protein–ligand docking and binding free energy calculations. The docking methods, which rely on efficient heuristic ligand placement searching algorithms and fast empirical scoring functions, offer the ability to predict quickly and cheaply the binding mode (pose) and affinity of a ligand for the protein receptor of interest and, therefore, are widely used in virtual screening of compound libraries and structure-based drug design [13,14]. Free energy calculations, which try to compute free energies of the protein–ligand systems based on the principles of statistical thermodynamics, are required to generate thermodynamic averages through extensive conformational sampling and, therefore, are more time-consuming approaches than the docking methods [3,15]. The advantages, disadvantages, and challenges of these methods will be discussed and/or compared.

## 2. Physicochemical Mechanisms of Protein–Ligand Interaction

In order to gain a deeper understanding of the molecular recognition between a protein and its ligand, it is necessary to understand the physicochemical mechanisms underlying the protein–ligand interaction. In this section, the binding kinetics, the basic thermodynamic concepts and relationships relevant to protein–ligand binding, and the binding driving forces/factors and enthalpy–entropy compensation, are introduced and/or rationalized.

### 2.1. Protein–Ligand Binding Kinetics

Protein–ligand binding kinetics describes the process underlying the association between the protein and ligand, particularly focusing on the rate at which these two partners bind to each other. In a simple instance, when a protein molecule P and a ligand molecule L with mutual affinity are mixed in a solution, the time-dependent association between them can be formulated as:

(1) $$P+L\overset{k_{\mathrm{on}}}{\underset{k_{\mathrm{off}}}{⇌}}\text{ PL}$$

where PL represents the protein–ligand complex, k_on_ and k_off_ are the kinetic rate constants that account for the forward binding and reverse unbinding (or dissociation) reaction, respectively. The units of k_on_ and k_off_ are M^−1^·s^−1^ and s^−1^, respectively. At equilibrium, the forward binding reaction P + L → PL should be balanced by the reverse unbinding reaction PL → P + L, and this is written:

k_on_[P][L] = k_off_[PL] (2)

where the square brackets represent the equilibrium concentration of any molecular species. The binding constant K_b_ (in unit of M^−1^) is then defined by:

(3) $$K_{b}=\frac{k_{\mathrm{on}}}{k_{\mathrm{off}}}=\frac{\text{[PL]}}{\text{[P][L]}}=\frac{1}{K_{d}}$$

where K_d_ (in unit of M) is called dissociation constant. Therefore, the fast binding rate accompanied by a slow dissociation rate will give a high/low binding/dissociation constant and, hence, a high binding affinity.

### 2.2. Basic Concepts and Thermodynamic Relationships

A protein–ligand–solvent system is a thermodynamic system composed of the solute (*i.e*., the protein and ligand molecules) and the solvent (*i.e*., liquid water and buffer ions). In such a system, there are very complex interactions and heat exchange among these substances; and the relationship between these substances and how heat transfer is related to various energy changes are dictated by the laws of thermodynamics. As a result, the driving forces that dictate the association between protein and ligands are a synthetic result of various interactions and energy exchanges among the protein, ligand, water, and buffer ions. Gibbs free energy, which is a thermodynamic potential that measures the capacity of a thermodynamic system to do maximum or reversible work at a constant temperature and pressure (isothermal, isobaric), is one of the most important thermodynamic quantities for the characterization of the driving forces [15,16]. In analogy with any spontaneous process, protein–ligand binding occurs only when the change in Gibbs free energy (Δ*G*) of the system is negative when the system reaches an equilibrium state at constant pressure and temperature. Because the protein–ligand association extent is determined by the magnitude of the negative Δ*G*, it can be considered that Δ*G* determines the stability of any given protein–ligand complex, or, alternatively, the binding affinity of a ligand to a given acceptor. It should be noted that the free energy is a function of the states of a system and, as thus, Δ*G* are defined merely by the initial and final thermodynamic states, regardless of the pathway connecting these two states.

The standard binding free energy Δ*G*°, which refers to the free energy change measured under the conditions of 1 atm pressure, a temperature of 298 K, and the effective reactant (protein and ligand) concentrations of 1 M, is related to the binding constant K_b_ by the Gibbs relationship:

Δ*G*° = −R*T*lnK_b_ (4)

where R is the universal gas constant (1.987 cal·K^−1^·mol^−1^) and *T* is the temperature in degrees of Kelvin. Equation (4) makes it apparent that the higher the binding constant K_b_, the more negative the standard free energy of binding, indicating that the kinetic parameters (k_on_ and k_off_ and their ratio K_b_) determine the thermodynamic properties of the complex, *i.e.*, the stability of the complex and the binding affinity between the protein and ligand.

The binding free energy (Δ*G*) at any moment in time during an association (not necessarily at standard-state condition) is given by:

Δ*G* = Δ*G*° + R*T*ln*Q* (5)

where the *Q* is the reaction quotient, which is defined as a ratio of the concentration of the protein–ligand complex to the product of the concentrations of the free protein and free ligand at any moment in time. When *Q* = K_b_ (as shown by Equation (3)), an association reaction is at equilibrium, and Δ*G* = 0. Δ*G* can also be parsed into its enthalpic and entropic contributions with the following fundamental equation:

Δ*G* = Δ*H* − *T*Δ*S* (6)

where Δ*H* and Δ*S* are change in enthalpy and entropy of the system upon ligand binding, respectively, and *T* is the temperature in Kelvin.

Enthalpy is a measure of the total energy of a thermodynamic system, *i.e.*, the sum of the internal energies of the solute and solvent and the amount of energy required to make room for the system (calculated as the product of the system volume and the pressure) [17]. Δ*H* is negative and positive in the exothermic (*i.e*., formations of the energetically favorable noncovalent interactions between atoms) and the endothermic (*i.e.*, disruptions of the energetically favorable noncovalent interactions) processes, respectively. For a binding process, Δ*H*, or the binding enthalpy, reflects the energy change of the system when the ligand binds to the protein. The binding enthalpy in a non-strict sense is generally treated as the changes in energy resulting from the formations of noncovalent interactions (van der Waals contacts, hydrogen bonds, ion pairs, and any other polar and apolar interactions) at the binding interface. However, the heat effect of a binding reaction is a global property of the entire system, including contributions not only from the solute, but also from the solvent [18], and it is barely conceivable to form favorable interactions without disrupting any others [6]. In fact, the change in enthalpy upon binding is a result of forming and disrupting many individual interactions, including the loss of the hydrogen bonds and van der Waals interactions formed between the protein and solvent and between the ligand and solvent, the formation of the noncovalent interactions between the protein and ligand, and the solvent reorganization near the complex surfaces. These individual components may make either favorable or unfavorable contributions, and the net enthalpy change is a result of the combination of these contributions [6,19].

Entropy is a measure of how evenly the heat energy will be distributed over the overall thermodynamic system. The second law of thermodynamics determines that the heat always flows spontaneously from regions of higher temperature to regions of lower temperature. This reduces the degree of the order of the initial system, and, therefore, entropy could also be viewed as a measure of the disorder or randomness in atoms and molecules in a system. Δ*S* is a global thermodynamic property of a system, with its positive and negative signs indicating the overall increase and decrease in degree of the freedom of the system, respectively. The total entropy change associated with binding (the binding entropy Δ*S*) may be parsed into three entropic terms:

Δ*S* = Δ*S*_solv_ + Δ*S*_conf_ + Δ*S*_r/t_ (7)

where Δ*S*_solv_ represents the solvent entropy change arising mainly from surface burial that results in solvent release upon binding, which often makes a favorable contribution to the binding entropy due to its large positive value; Δ*S*_conf_ represents the conformational entropy change reflecting the changes in the conformational freedom of both the protein and ligand upon binding, which may contribute favorably or unfavorably to the binding entropy because the degree of freedom of the complex may increase or reduce as compared to those of the unbound, free protein and ligand [20,21]; Δ*S*_r/t_ represents the loss of translational and rotational degrees of freedom of the protein and ligand upon complex formation, which reduces the number of particles in solution and contributes unfavorably to the binding entropy. The above three entropic terms determine the net entropy change, with positive and negative net entropy change contributing favorably and unfavorably to the binding free energy, respectively. Generally, the binding reactions would have to overcome the inescapable entropic penalties (*i.e.*, the negative Δ*S*_r/t_ upon binding) [22,23] through either large solvent entropy gain (positive Δ*S*_solv_) or favorable protein–ligand interactions (which lead to negative binding Δ*H*) if binding is to occur [19].

### 2.3. Binding Driving Forces and Enthalpy-Entropy Compensation

Because (i) only when the change of the system free energy is negative can the protein–ligand binding occur spontaneously; and (ii) the extent of the difference in free energy between the complex state and the unbound free state (*i.e*., the magnitude of the negative free energy change upon binding) determines the stability of the complex, it can be considered that it is the decrease in system free energy that drives the protein–ligand binding. In fact, both the protein folding and protein–ligand binding processes are driven by the decrease in total Gibbs free energy of the system. The only difference between them is the presence and absence of the chain connectivity, which leads to two different terms: intramolecular and intermolecular recognition and binding [24,25]. However, the common driving forces and similar folding/binding processes have led to similar free energy funnel models (folding funnel and binding funnel) for explaining these two similar processes [26,27,28,29].

As introduced above, two thermodynamic quantities, the enthalpy change and entropy change, determine the sign and magnitude of the binding free energy. We therefore consider Δ*H* and Δ*S* as the driving factors for protein–ligand binding. The contributions of Δ*H* and Δ*S* to Δ*G* are closely related. For instance, the tight binding resulting from multiple favorable noncovalent interactions between association partners will lead to a large negative enthalpy change, but this is usually accompanied by a negative entropy change due to the restriction of the mobility of the interacting partners, ultimately resulting in a medium-magnitude change in binding free energy [30]. Similarly, a large entropy gain is usually accompanied by an enthalpic penalty (positive enthalpy change) due to the energy required for disrupting noncovalent interactions. This phenomenon—the medium-magnitude free energy change caused by the complementary changes between enthalpy and entropy—is called the enthalpy–entropy compensation.

It should be noted that this phenomenon has been a subject of debate for decades. The main criticisms are that the compensation could be (i) a misleading interpretation of the data obtained from a relatively narrow temperature range [31] or from a limited range for the free energies [32,33]; (ii) the result of random experimental and systematic errors [34,35]; and (iii) the result of data selection bias [36,37,38]. Nevertheless, enthalpy–entropy compensation has been very frequently observed in thermodynamic binding studies of biological systems [6,21,39,40,41], and analyses of collected calorimetric data for protein–ligand binding [36,42,43] and results from theoretical studies [44,45] suggest that it is a genuine and common physical phenomenon, although stringent criteria for the assignment of true compensation effects must be adhered to. The enthalpy–entropy compensation may be rooted in the formations and disruptions of the weak noncovalent interactions. Multiple factors seem to influence the compensation behavior, including the structural and thermodynamic properties of the solvent (hydrophobic effect, solvation, desolvation, and local water structure), the flexibility of the ligand-binding site/pocket or of the regions in the surrounding of the localized site, the molecular structure of the ligand, and the changes in intermolecular forces during the binding process [30,44,46,47,48,49]. In addition, the mechanisms of entropy–enthalpy transduction [42] have been proposed to explain entropy–enthalpy compensation.

Because the enthalpy-entropy compensation does not give rise to dramatic change in the binding free energy, the discrimination between the entropic and enthalpic contributions to the binding free energy is important in the fields of medicinal chemistry and rational drug design. The ideal optimization strategy is to maximize the favorable enthalpic or entropic contribution while minimizing the entropic or enthalpic penalty. The ultimate goal is to induce the largest decrease in binding free energy, thereby defeating the deleterious effects of the enthalpy–entropy compensation at the thermodynamic level [6].

## 3. Protein–Ligand Binding Models

**Figure 1:**
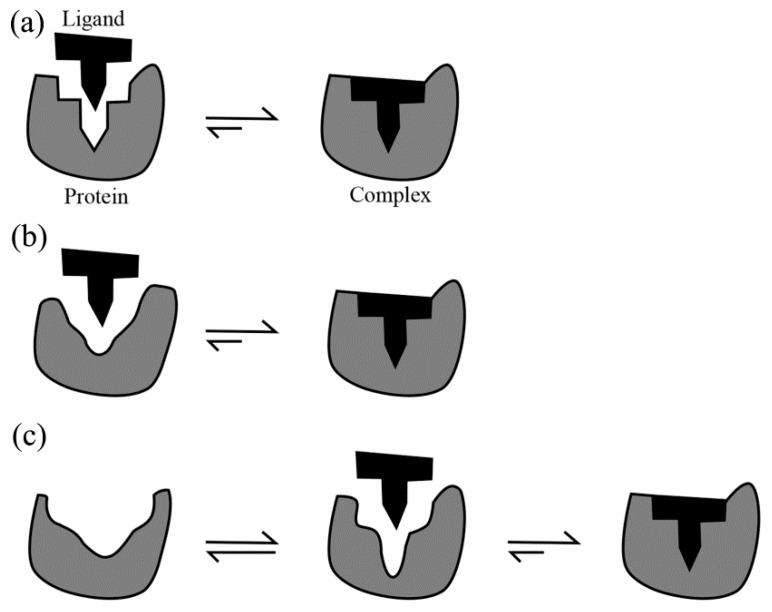
Schematic illustrations of the three protein-ligand binding models: (**a**) Lock-and-key; (**b**) Induced fit; and (**c**) Conformational selection. Adapted from [52].

Three different models, the “lock-and-key” [50], “induced fit” [51] and “conformational selection” [24,26,52,53], have been proposed to explain the protein–ligand binding mechanisms. The prerequisites of the lock-and-key model (Figure 1a) are that both the protein and the ligand are rigid and their binding interfaces should be perfectly matched. As a result, only the correctly sized ligand (the key) can insert into the binding pocket (key hole) of the protein (the lock). However, the lock-and-key model cannot explain the experimental evidence that a protein binds its ligand when their initial shapes do not match well. This leads to the induced fit model (Figure 1b), which assumes that the binding site in the protein is flexible and the interacting ligand induces a conformational change at the binding site. Because the induced fit mechanism takes into account only the conformational flexibility of the ligand-binding site, this model seems to be suitable for proteins showing merely minor conformational change after the ligand binding. In addition, both the lock-and-key and the induced fit models treat the protein as a single, stable conformation under given experimental conditions. However, most proteins are inherently dynamic and the conformational selection model takes into account this inherent flexibility. The conformational selection model (Figure 1c), which derives from the free energy landscape (FEL) theory of protein structure and dynamics [54,55,56,57], postulates that the native state of a protein does not exist as a single, rigid conformation but rather as a vast ensemble of conformational states/substates that coexist in equilibrium with different population distributions, and that the ligand can bind selectively to the most suitable conformational state/substate, ultimately shifting the equilibrium towards this state/substate. In other words, the unbound protein can sample with a certain probability the same conformation as that of the ligand-bound state.

The underlying mechanisms of these three models and relevant case studies have been extensively reviewed elsewhere [5,53,58,59,60]. As a supplement, we here mainly focus on discussing how the protein–ligand binding processes are driven/dominated by the enthalpy and/or entropy changes under the scheme of the three models. This will facilitate having an in-depth understanding of the binding mechanisms and could help to improve binding affinity by modifying the acceptor or the ligand.

### 3.1. Diffusion Followed by Collision Is the Prerequisite for Binding

For binding to proceed, the initial contacts/collisions between a protein molecule and a ligand have to occur to form an encounter complex, for which the molecular diffusion plays a decisive role [61]. Molecular diffusion, which originates from molecular kinetic energy (or heat, thermal energy), is an entropy-driven process [17]. In a protein–ligand–solvent system, the diffusion (or the random Brownian motions) of solute molecules have two origins: (i) the kinetic energy of the solute molecules themselves; and (ii) collisions of the large solute with the small water molecules, which move with different velocities in different random directions. At the constant temperature and pressure, the motions of individual water molecules resulting from their atomic kinetic energy could lead to the maximization of the solvent entropy. On the other hand, it seems likely that the heavy Brownian bombardments from a large amount of water molecules may play a role in facilitating the rotations, translations, and wanderings of the solute molecules and, ultimately, the accidental contacts/collisions between them [62].

It should be noted that the long-range electrostatic attraction can speed up the association between two solute molecules with opposite charges and as thus overcomes the diffusion limit [63]. A quantitative description of the biomolecular diffusion-collision can be found in [61] and references therein. The Brownian dynamics (BD) simulation approach can be used to predict diffusion-controlled association rates and can provide information about association pathways that lead from a freely diffusing ligand towards a protein–ligand encounter complex [64,65,66].

### 3.2. Lock-and-Key: An Entropy-Dominated Binding Process

Using geometric-complementary colloidal particles as study objects, the lock-and-key binding and the underlying mechanisms have been investigated experimentally [67,68] or theoretically by means of density functional theory [69], hypernetted-chain integral equation theory [70,71], dissipative particles dynamics [72], and Monte Carlo (MC) simulations [73,74]. A common conclusion is that the depletion force [75] originating from the overlapping exclusion volume effect [67], or the depletant/solvent entropy maximum [74,76], rules the interaction between lock-key colloids. In other words, the lock-and-key binding between colloidal particles is entropically driven, either in the absence of attractive forces [73] or even in the presence of electrostatic repulsion [67,74], and it depends only on the size, shape, or surface roughness of interacting colloids (*i.e*., how well the lock and key surfaces match), regardless of composition and surface chemistry [77,78]. Based on these studies, below we will give a tentative rationalization of the lock-and-key binding process and its driving forces.

It seems likely that the initial collisions occurring between the complementary interfaces of the protein and ligand would trigger the displacement of the water molecules surrounding the solute molecules, which leads to an increase of the accessible volume for the solvent molecules, ultimately causing a drastic increment of the solvent entropy. Prior to the collision, water molecules formed a well-defined network (or hydration shell) around the surfaces of the solute molecules [79]. This solvation process, on the one hand, leads to a negative enthalpy change due to the formation of the hydrogen-bonding and van der Waals interactions between the solvent and the solute and between different water molecules, and, on the other hand, results in the decrease in solvent entropy due to the loss of the degrees of freedom of the hydrated water molecules. The initial collision between the protein and the ligand may disrupt some of the noncovalent interactions within the water network and, hence, results in the release of a fraction of water molecules in the water network. The energy required for disrupting the favorable interactions (positive enthalpy change) comes from the molecular kinetic energy, while the release of the constrained water increases the solvent entropy [74]. The perfectly matched interfaces between the protein and the ligand under the key-and-lock model make it possible for the initial collision to trigger a complete displacement of the water networks surrounding the interaction interfaces, thus producing a large amount of the solvent entropy. In addition, under the rigid hypothesis, there is no change in the conformational entropy. Therefore, for the lock-and-key binding to proceed, the solvent entropy gain should be large enough to overcompensate for not only the positive enthalpy change arising from the desolvation process, but also the negative entropy change caused by the loss of rotational and translational motions of the ligand.

Indeed, the negative enthalpy change arising from the favorable interactions (such as van der Waals forces, hydrogen bonding, electrostatic, and dipole–dipole interactions) can also contribute to the lowering of the system’s free energy, but the solvent entropy gain arising from the displacement of the water molecules plays a dominant role in lowering the free energy [74,80]. Therefore, it is reasonable to conclude that the lock-and-key binding is a entropy-dominated process.

### 3.3. Induced Fit

Typical induced fit binding has been demonstrated in the designed host-guest systems [45,81]. Through dynamic combinatorial chemistry method, Otto *et al.* discovered a receptor termed diastereomer 4 that binds to its ligand NMe4I via the induced fit mechanism [81]: the evidence for the induced fit recognition comes from the NMR study of the receptor; the ITC data indicate that this binding is strongly enthalpy driven (Δ*G*° = −9.1 kcal·mol^−1^, *T*Δ*S*° = −0.2 kcal·mol^−1^, Δ*H*° = −9.3 kcal·mol^−1^). Using the second-generation mining minima algorithm [82], Chang & Gilson found that bindings of two ligands (termed barbital 11 and phenobarbital 12) to a flexible macrocyclic barbiturate receptor 10 are involved in the induced fit mechanism [45]: the formation of six strong hydrogen bonds between binding partners induces restructuring of the receptor 10 to form a complementary binding site; the negative enthalpy changes are large enough to overcompensate for the strongly unfavorable losses in conformational entropy, thus achieving more negative binding free energies (by greater than −4.0 kcal·mol^−1^) than observed in another complex (receptor 10-mephobarbital 13), in which the lack of strong interactions between mephobarbital 13 and the receptor 10 prevents the induced fit seen for the two ligands barbital 11 and phenobarbital 12. With regard to the protein–ligand system, the evidences from X-ray crystallographic structures suggest that the sugar binding to permease is likely involved in the induced fit process [83,84,85]. Furthermore, the ITC measurement shows that the binding of nitrophenyl-α-galactoside to the sugar permease MelB from Salmonella typhimurium is solely driven by favorable negative enthalpy change (−10.3 kcal·mol^−1^), which overcompensates for the unfavorable negative entropy change (−3.8 kcal·mol^−1^) and, hence, dominates the induced fit binding [86]. These studies, in conjunction with the kinetic model calculations demonstrating that binding by induced fit makes sense only if there is a certain extent of pre-existing complementarity between the interacting species [87], allow us to rationalize tentatively the induced fit binding process and its driving forces, as described below.

For the binding to take place under the induced fit model, the lack of perfect surface complementary between binding partners necessitates multiple tentative collisions to achieve an appropriate match between the interacting sites [17,87]. The initially established contacts (negative enthalpy change) between the matched sites should be strong enough to provide the encounter complex enough strength and longevity so that induced fit takes place within a reasonable time [87]. In addition, the amount of the released constrained water molecules upon encounter complex formation, although smaller than that in the lock-and-key model due to the imperfectly matched interacting sites, can also make a favorable contribution to the stability of the encounter complex. The subsequent induced fit is in essence a process of adjusting conformation of the binding site to suit the needs of the incoming ligand, ultimately leading to maturation of the encounter complex into a fully bound complex. This process is also accompanied by the release of the water molecules and, moreover, because of the excellent shape match between the binding partners in the fully bound complex, the amount of released water in the overall process of the induced fit binding can be expected to be as much as that of the lock-and-key binding. As a result, the solvent entropy gain also contributes favorably to the induced fit binding. Nevertheless, the net entropy change of binding is determined by the three entropic terms, *i.e*., Δ*S*_solv_, Δ*S*_conf_, and Δ*S*_r/t_, as shown in Equation (7). In the case of the induced fit binding, it can be speculated that the Δ*S*_conf_ term is negative since the formed favorable noncovalent interactions between the binding partners restrict the conformational freedoms of the interacting interfaces. Such an unfavorable Δ*S*_conf_ term, together with the unfavorable (negative) Δ*S*_r/t_ term (due to the loss of rotational and translational degrees of freedom of the binding partners), tends to compensate for the favorable (positive) Δ*S*_solv_ term, ultimately leading to a relatively small net entropy change compared to the net enthalpy change. Indeed, in the examples of induced fit binding [45,81,86] described above, the net entropy changes are all negative values, which are, however, overcompensated for by the relatively larger negative enthalpy changes. It is not impossible that the net entropy change could be a positive value and contributes favorably to the lowering of the system’s free energy, but the magnitude of the net negative enthalpy change is larger, thus contributing substantially to the binding free energy in the induced fit binding.

In the induced fit process, it is hard to imagine that the new noncovalent interactions between the protein and the ligand will be formed without disrupting any original interaction at the binding sites. In order to maintain the stable association of binding partners in the maturation complex, the negative enthalpy change resulting from the newly established interactions should be large enough to overcompensate for not only the positive enthalpy change resulting from disrupting the original interactions, but also the possible negative net entropy change. Intuitively, the magnitude of the negative enthalpy change could be related either to the number of noncovalent interactions, or to their strength, between the protein and the ligand. Taken together, it is not unreasonable to consider that the induced fit binding is dominated by the enthalpic term [21], or alternatively, that it is an enthalpy-dominated process [17].

### 3.4. Conformational Selection: A Process in Which Entropy and Enthalpy Play Roles in a Sequential Manner

The conformational selection model is based on the FEL theory and explains the binding events by considering the population distribution and redistribution of protein conformational states/substates [59,60]. First, the distribution of the conformational states/substates of protein molecules with different probabilities at the rugged bottom of the funnel-like FEL allows for the selective interaction of a ligand with the conformational state/substate that has the shape of the binding site best matching the ligand [24,26,88,89]. This step may not “induce” a conformational change and is similar to the binding process described by the lock-and-key model [59]. Therefore, the selective binding may be dominated by the solvent entropy gain. Second, the presence of the conformational flexibility in the protein allows for the conformational adjustments of the residue side chains or even of the backbone to form the strong intermolecular noncovalent interactions [53,90,91]. This step is similar to the binding process described by the induced fit model and as such may be dominated by the system enthalpy decrease. Third, the formation of the protein–ligand complex alters the heights of the free energy barriers (or the conformational transition rates) that separate the bound state/substate and its adjacent states/substates, thus resulting in a population shift towards the bound state/substate and the conformational redistribution [59,92,93,94].

For the conformational selection binding scenario, it is difficult to distinguish which factor (the entropy or the enthalpy) contributes more to the lowering of the system’s free energy because the large solvent entropy gain in the first step could be offset by the loss of the rotational and translational entropy and the decrease of the conformational entropy in the subsequent step, and the negative enthalpy change in the second step could be offset by the positive enthalpy changes due to the desolvation energy penalty and the disruption of the original noncovalent interactions surrounding the binding sites. Nevertheless, the selective binding and the following conformational adjustments are dominated by the solvent entropy gain and the system enthalpy decrease, respectively, suggesting that they play a role, in a sequential manner, in lowering the system’s free energy. In addition, the conformational selection model takes into account the distribution and redistribution of the populations of protein conformational states/substates, which allow a protein to interact with multiple structurally distinct binding partners and accommodate mutations through shifts in the dynamic FEL, and, as such, is evolutionarily advantageous [19,89,95].

### 3.5. The Relationship between Lock-and-Key, Induced Fit and Conformational Selection

Under the background of the funnel-like FEL, the lock-and-key may be viewed as an “extremity” of the conformational selection [19,59]. A high-rigidity protein has a very smooth folding funnel where there is no ruggedness around the bottom of the funnel, thus resulting in only one conformer that occupies a single free energy well in the global free energy minimum region. A high-flexibility protein has multiple free energy minima (or wells) within which ensembles of different conformational states/substates are located. However, the conformational selection model assumes that the selective binding occurs only in one free energy well that contains the most suitable conformer for binding, thus resembling the lock-and-key binding occurring in the single, global free energy minimum well. The difference between these two models is that conformational selection induces a population shift and the redistribution of the states/substates, whereas the population shift cannot be presented in the lock-and-key model [60]. Another difference between these models, as proposed by Nussinov *et al.* is the “selected object” [60], which is a conformer out of many different conformers in the ensemble of the same protein for the conformational selection model and a protein out of many different proteins for the lock-and-key model. As a result, they suggested that the lock-and-key mechanism addressed the question of which protein—out of the many in the cell—will be bound by a given ligand [60].

The induced fit involved in the conformational adjustments is a key step in the conformational selection mechanism, and the enhanced interactions resulting from this step could accelerate the population shift, implying that induced fit can extend and optimize conformational selection [60,96]. For the binding process to proceed in the classical induced fit mechanism, the selective initial interactions must be strong enough to maintain the encounter complex for a relatively long time [87], which indicates that induced fit also contains the step of selecting the appropriate initial conformation [17,19] or, alternatively, the “conformational selection” plays a role in induced fit.

For a ligand to bind to a given flexible protein, there has been much debate as to whether the conformational selection or the induced fit is the governing mechanism. Hammes *et al.* have proposed a flux-based method by which the sequence of events during binding can be determined quantitatively [97]. Applications of this method to two examples—binding of NADPH to dihydrofolate reductase and flavodoxin folding coupled to binding of flavin mononucleotide—revealed that in both cases the binding mechanism switches from being dominated by the conformational selection pathway at a low ligand concentration to the induced fit at a high ligand concentration. More recently, using the induced fit fraction index (*i.e*., the fraction of binding events achieved via induced fit) to quantify the binding mechanism, Greives and Zhou demonstrated that the conformational selection dominates at the slow conformational transition of the protein and the low ligand concentration, while the induced fit dominates when either quantity is increased [98]. Through establishing a solvable model of active–inactive conformational transitions for the protein, Zhou also showed that, as the active-inactive transition rates increase, the binding mechanism gradually shifts from the conformational selection to the induced fit [99]. The above results point to the conclusion that both the ligand concentration and the timescale of protein dynamics play a role in shifting the binding mechanism between the conformational selection and induced fit.

Since all three distinct conceptual models have been observed experimentally, it is important to keep in mind that all three mechanisms may exist both in a simultaneous or in a sequential manner, covering a broad spectrum of binding events [100].

## 4. Methods Used to Investigate Protein–Ligand Binding Affinity

### 4.1. Experimental Methods

Many experimental techniques can be used to investigate various aspects of protein–ligand binding. X-ray crystallography, nuclear magnetic resonance (NMR), Laue X-ray diffraction, small-angle X-ray scattering, and cryo-electron microscopy provide atomic-resolution or near-atomic-resolution structures of the unbound proteins and the protein–ligand complexes, which can be used to study the changes in structure and/or dynamics between the free and bound forms as well as relevant binding events. For example, X-ray-diffraction data contain information not only about the enthalpic contribution (intermolecular noncovalent interactions) but also about some entropic contribution (spatial distribution around the average structure as reflected by the B-factors) [21,55]; NMR methods have the advantage of characterizing the protein–ligand dynamics over a wide range of timescales from picoseconds to seconds [101] and, hence, are powerful for investigating entropic contribution to the binding free energy [102]; Laue X-ray diffraction can measure simultaneously the structures and kinetics, with the added advantage of delivering the timescale of local motions [103]; and cryo-electron microscopy and small-angle X-ray scattering can determine directly the structural ensemble with relatively low resolution in the experimental conditions, although they cannot characterize the timescales of conformational transition [55]. Other experimental methods that have been applied to study the protein dynamics involved in binding include single-molecule fluorescence spectroscopy [104] and time-resolved hydrogen-deuterium exchange mass spectrometry [105].

Three groups of methods deserving special attention in the context of protein–ligand binding affinity include ITC [6,7,8], SPR [9,10,106], and FP [11,12,107], which will be introduced and discussed in detail in the following.

#### 4.1.1. Isothermal Titration Calorimetry (ITC)

The structural and dynamic data alone, even when coupled with the most sophisticated computational methods, cannot provide information about the complete thermodynamic profiles consisting of the binding free energy, entropy, and enthalpy, and, therefore, may not accurately predict the binding affinity [7]. However, the calorimetric techniques, including the differential scanning calorimetry (DSC) and the ITC, provide quantitative thermodynamic data that can be used to study the complex stability and to elucidate the binding driving forces. DSC can measure the enthalpy and the heat capacity of thermal denaturation and as thus provides a way to estimate the stability of protein–ligand complexes [108,109]. ITC is the only approach to measure directly the heat exchange during complex formation at a constant temperature, and has become the gold standard in determining the forces that drive the binding process or stabilize the intermolecular interactions [6,21]. Next, the ITC method and its applications will be introduced in more detail.

##### Experimental Setup, Principles, and Data Processing of ITC

A typical ITC experiment contains three steps: (i) a ligand is titrated into a solution containing the bio-macromolecule (e.g., protein) of interest; (ii) the heat released or absorbed that is associated with a binding event is measured; and (iii) the primary ITC data is processed and fitted to obtain the binding constant (K_b_), Gibbs free energy of binding (Δ*G*), binding enthalpy (Δ*H*) and entropy (Δ*S*), and the stoichiometry (n) of the binding event [110]. Moreover, when ITC experiments are performed at varying temperatures, the heat capacity change (Δ*C*_p_) of a binding reaction can be obtained.

ITC instruments make use of a power compensation design, which is responsible for maintaining the same temperature in the sample cell containing the macromolecule and the reference cell filled with buffer or water. During the experiment, a titration system delivers the ligand to the sample cell in precisely known aliquots; this causes heat to be either released or absorbed (depending on the nature of the reaction) and, hence, a temperature imbalance between the reference and sample cell. Such an imbalance is compensated for by modulating the feedback power applied to the cell heater: exothermic and endothermic reactions decrease and increase the power to the sample cell, respectively. The overall measurements consist of the time-dependent input of the power required to maintain equal temperatures between the sample and the reference cells at each titration.

**Figure 2:**
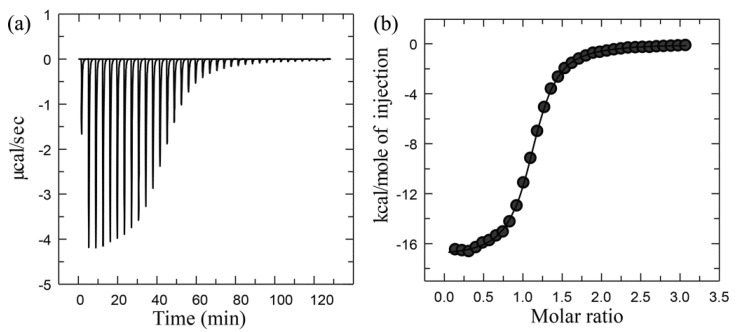
Representative ITC data for the binding of cytidine 2′-monophosphate (2′CMP) to RNaseA: (**a**) Primary raw data; (**b**) Binding curve derived from the raw data. Adapted from [7] Copyright 2008 with permission from Annual Reviews.

Figure 2 shows the representative ITC data. The primary data is the power applied to the sample cell as a function of time, and consists of a series of peaks that return to the baseline, with the area under each peak corresponding to the heat evolved at each ligand injection (Figure 2a). As the ligand-binding site becomes gradually saturated, the magnitude of the peak area decreases gradually until only heat of dilution is observed. The binding curve (the form of a binding isotherm), which is obtained via transformation of the primary ITC data, represents the heat of reaction per injection as a function of the ratio of the total ligand concentration to the protein concentration [L]/[P] (Figure 2b). Finally, fitting the binding curve to a particular binding model can yield the parameters K_b_, Δ*H*, and *n* from a single experiment. The models for fitting ITC data can be found in [6,7,111] and references therein. The binding constant K_b_ can be used to calculate the standard binding free energy Δ*G*° according to Equation (4); the binding free energy Δ*G* can be calculated from Δ*G*° according to Equation (5); and the binding entropy Δ*S* can be calculated according to Equation (7). The Δ*C*_p_, which is a thermodynamic quantity that measures the change in heat with temperature at a constant pressure, can be obtained through determining Δ*H* values at a range of temperatures with ITC followed by calculating the slope of the temperature-dependent Δ*H* curve with linear regression analysis. Δ*H*, Δ*S*, and Δ*G* are dependent on the temperature through Δ*C*_p_:

Δ*H*(*T*) = Δ*H*(*T*_0_) + Δ*C*_p_(*T* − *T*_0_) (8)

(9) $$\Delta S\left(T\right)=\Delta S\left(T_{0}\right)+\Delta C_{p}\text{ln}\left(\frac{T}{T_{0}}\right)$$

(10) $$\Delta G(T)=\Delta H(T_{0})-T\Delta S(T_{0})+\Delta C_{p}\left(T-T_{0}-T\text{ln}\frac{T}{T_{0}}\right)$$

where *T* and *T*_0_ refer to the temperature and an appropriate reference temperature, respectively.

Because there is strong correlation between Δ*C*_p_ and the surface area buried on forming a complex, Δ*C*_p_ provides a link between thermodynamic parameters and the structural information of proteins [6,112]. The hydrated water (particularly those interacting with the hydrophobic surface) and the bulk water have different behaviors and properties; this will lead to a change in heat capacity due to the release of water molecules upon complex formation, with the magnitude of Δ*C*_p_ being proportional to the amount of surface area involved. The desolvation of both the protein and ligand upon binding can make positive or negative contribution to Δ*C*_p_, depending on the burial of the apolar (leading to negative Δ*C*_p_) or polar (positive Δ*C*_p_) surface areas [6,113].

If the concentrations of both the protein and the ligand are known, ITC can determine the binding stoichiometry (n) from the molar ratio of the interacting partners at the equivalence point. In data fitting, the parameter n can either be fixed as the number of binding sites per macromolecule or be treated as an additional floating parameter determined from iterative fitting [6]. Proteins with more than two interacting sites often involve multiple binding events and play an important role in regulating biological system. Therefore, the determination of n is of central importance in characterizing their binding mechanisms and, further, in understanding the relevant biological processes.

It is important to keep in mind that the heat exchange detected by the ITC experiment is the total heat effect in the sample cell upon addition of the ligand, including not only the heat absorbed or released during binding reactions, but also the heat effects arising from dilution of the ligand and protein, mixing two solutions containing different compositions, temperature differences between the sample cell and the syringe, and so forth. As a result, control experiments need to be performed to determine these non-specific heat effects to obtain the heat of complex formation.

##### Advantages and Disadvantages of ITC

In addition to its ability to determine basic thermodynamic parameters (enthalpy, binding constant, entropy, and stoichiometry) from a single titration, ITC has the following advantages as compared to other biophysical techniques: (i) a non-destructive technique because the thermodynamic parameters of the interaction can be measured in solution without immobilization, modification, or labelling of the binding partners and without molecular weight restrictions [114]; (ii) the high precision and reproducibility, with error of determined binding constant being typically in the range of 5% [115]; (iii) the high robustness and sensitivity, with ability to measure binding affinities in the ranges of ~10^−2^–10^3^ μM, heat effects as small as 0.1 μcal, and heat rates as small as 0.1 μcal/s [116]; and (iv) the ability to measure high affinity interactions with binding constant as large as 10^8^–10^9^ M^−1^ [116,117,118].

However, there are still certain disadvantages to ITC. Since heat is a universal signal and each process contributes to the measured global heat effect, the evaluation of the contribution from the binding is complicated. Despite the high sensitivity, challenges still exist for extracting heat effects of complex formation when the binding exhibits rather small binding enthalpy (resulting in relatively low signal to noise) and when the binding processes are very slow (leading to the neglect of kinetically low processes). ITC generally needs a large amount of sample, which limits its application to certain bio-macromolecules that are difficult to prepare in large quantities. Traditional ITC is categorized as a method that is laborious, time-consuming, and low throughput, making it not very suitable for biotechnological and pharmaceutical applications that require low labor intensity and high throughput [7,119]. With the development of the modern ITC instruments by MicroCal (Worcestershire, UK) and Calorimetry Sciences Corporation (Lindon, UT, USA), this situation is now changing. These robotic automated instruments realize cell loading and data collection from large numbers of samples in an almost autonomous fashion, revolutionizing the way that ITC is employed in high-throughput research. In addition, the developments in the form of array-based nanocalorimeters allow parallel enthalpy measurements for a true high-throughput screening (HTS).

##### Case Study Using ITC

As a powerful tool for characterizing interactions of biomolecules in a broad range of binding affinities, ITC has gained wide applications in fields of biophysics, biochemistry, drug discovery, design and development, protein engineering, biotechnology, *et al.* Various applications of ITC have been reviewed in a series of papers to which the readers are referred for further details [114,115,117,119,120,121]. Here, we select one example to demonstrate the power of ITC in optimizing the affinity of drug candidates.

The affinity optimization of drug candidates is a major goal in drug development. In order to improve the binding affinity, the binding free energy between the lead compound and the target protein needs to be lowered through optimizing the enthalpic and/or entropic contributions to overcome the enthalpy-entropy compensation. The thermodynamic profile/signature (*i.e.*, changes in enthalpy, entropy, free energy, and heat capacity) measured by ITC provides clues for optimizations of these binding driving forces/factors [122].

**Figure 3:**
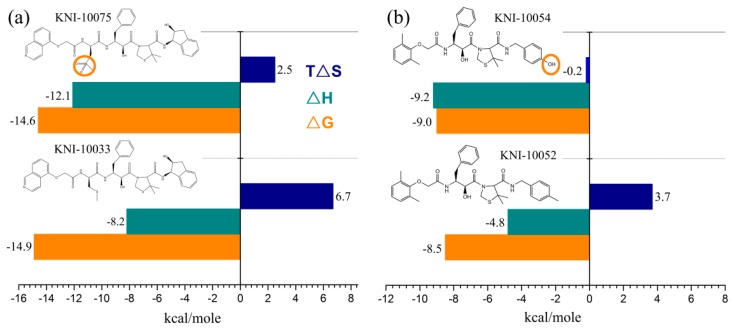

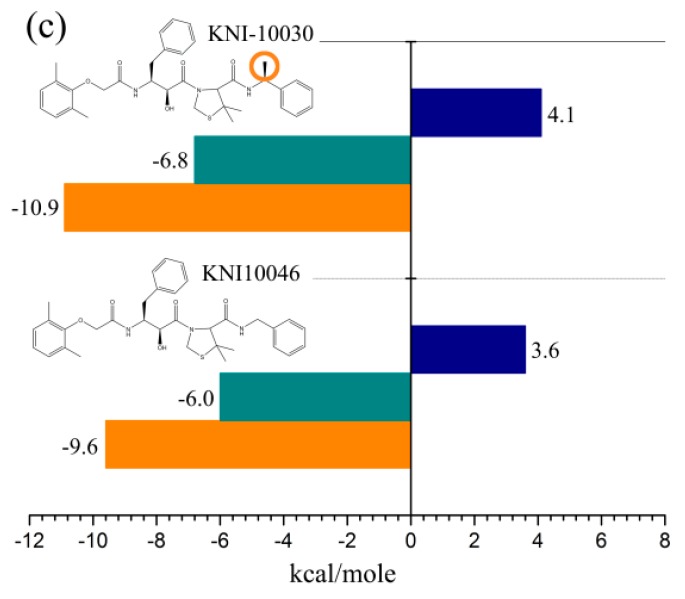
Thermodynamic profiles for three pairs of HIV-1 proteinase inhibitors that vary by only a single group: (**a**) KNI-10033-KNI-10075 pair within which an apolar group thioether on KNI-10033 is replaced by a polar group sulfonyl to form KNI-10075; (**b**) KNI-10052-KNI-10054 pair within which an apolar methyl group is replaced by a polar hydroxyl group; and (**c**) KNI-10046-KNI-10030 pair within which a hydrogen atom on the former is replaced by an apolar methyl group to form the latter. The binding free energy (Δ*G*), enthalpy (Δ*H*), and entropy (*T*Δ*S*) are shown. The data shown are taken from [122,127,128].

Freire laboratory has performed a series of studies to optimize HIV-1 proteinase inhibitor binding by consideration of the thermodynamics of the binding interactions [123,124,125,126,127,128]. Figure 3 shows the thermodynamic profiles for three pairs of HIV-1 proteinase inhibitors, with the difference in each pair being merely a single functional group. For the first pair [127] (Figure 3a), the replacement of a thioether on KNI-10033 by a sulfonyl on KNI-10075 results in a more negative enthalpy change (by −3.9 kcal·mol^−1^) but a less entropy gain (by −4.2 kcal·mol^−1^), thus ultimately elevating the binding free energy by 0.3 kcal·mol^−1^. These results indicate that although the addition of a single polar group introduces a strong hydrogen-bonding interaction with the target, the quantity of the decreased enthalpy cannot overcome that of the decreased entropy, resulting in a small rise in binding affinity. For the second pair [127] (Figure 3b), the replacement of a methyl group (KNI-10052) by a hydroxyl group (KNI-10054) results in a more negative enthalpy change of −4.4 kcal·mol^−1^, which is large enough to overcompensate for the entropic decrease (−3.9 kcal·mol^−1^), ultimately resulting in a slight lowering of the binding free energy (−0.5 kcal·mol^−1^). However, such a small free energy lowering corresponds to a large increase in binding affinity by a factor of 2 [122]. These results indicate that if the negative enthalpy change resulting from the introduction of a polar group is large enough, it is possible to overcome the entropic loss and increase the binding affinity. For the third pair [128] (Figure 3c), the addition of a methyl group to KNI-10046 results in a lower free energy of KNI-10030 (−10.9 kcal·mol^−1^) than its precursor (−9.6 kcal·mol^−1^). This is caused by more favorable enthalpy (by −0.8 kcal·mol^−1^) and entropy (by 0.5 kcal·mol^−1^) contributions of KNI-10030 compared to those of KNI-10046. These results indicate that the introduction of an apolar group brings about both the favorable changes in the enthalpy and entropy, thus resulting in 8.7-fold higher binding affinity but a more hydrophobic compound. Nevertheless, the highly hydrophobic compounds have been recognized to have problems with solubility, bioavailability, and selectivity [122]. On the contrary, the more hydrophilic compounds likely have better physicochemical parameters such as the lipophilicity and solubility that are necessary for favorable enthalpic binding [129]. Although the enthalpy-driven optimization is more challenging compared to the entropy-driven optimization, the development of enthalpically optimized compounds is becoming a trend for drug design [122,125,129].

#### 4.1.2. Surface Plasmon Resonance (SPR)

SPR, which is an optical-based method to measure the change in the refractive index near a sensor surface, is label-free and capable of measuring real-time quantification of protein–ligand binding kinetics and affinities [130]. SPR has been developed and performed predominantly using Biacore™ technology [131,132,133] (Uppsala, Sweden). In a Biacore instrument, the sensor surface is a thin film of gold on a glass support, which forms the floor of a flow cell through which an aqueous solution flows continuously. The protein receptor molecules are immobilized on the sensor surface, and the ligand (usually called the analyte molecule) is injected into the aqueous solution to detect the binding reaction. As ligands bind to immobilized receptor molecules, an increase in the refractive index (expressed in response units, RU) is observed. After a desired association time (*i.e.*, when all binding sites are occupied), a solution containing no ligand is injected through the flow cell to dissociate the protein–ligand complex. As the ligand dissociates from the immobilized protein, a decrease in RU is observed. The time-dependent RU curves can then be used to calculate the kinetic association rate constant k_on_ and the dissociation rate constant k_off_. The binding constant K_b_ can be obtained according to Equation (3).

The capacity of SPR to measure the real-time binding data makes it well suited to analyses of the binding kinetics, although the mass transport limitation makes it difficult to measure accurately the k_on_ value faster than ~10^6^ M^−^^1^·s^−^^1^ [134,135]. Compared to ITC, SPR has the ability to measure higher binding affinities, typically in the ranges of 10^−^^6^–10 μM [136]. For SPR, the highly reproducible affinity measurements, in conjunction with precise temperature control, make it possible to estimate binding enthalpy via van’t Hoff analysis [137], which, although not as rigorous as ITC, requires much smaller amounts of protein sample [134]. Although traditional SPR technique is not well suited to high-throughput assays [134], recent developments in SPR instrumentation, sensor chip design, and sample preparation strategies show that SPR has a high potential for HTS screening of membrane protein ligands [130,138]. It is important, however, to keep in mind that the protein immobilization affects the conformational and translational/rotational entropies, and therefore, the association rate [100].

#### 4.1.3. Fluorescence Polarization (FP)

Fluorescence has a wide spectrum of wavelengths, and, therefore, multiple colors can be applied for detecting the binding of the fluorescent-labelled ligand to a target. Fluorescence-based techniques used for investigating intermolecular interactions include fluorescence anisotropy [139], fluorescence correlation spectroscopy [140], time resolved fluorescence [141], FP [12], *etc.*

Among them, the fluorescence polarization has the capacity to measure the kinetics and thermodynamics of protein–ligand binding. The principle of FP derives from the fact that an initially polarized fluorescence emission becomes unpolarized over time, and this happens faster in the unbound than in the bound state [142,143]. FP can utilize the competition binding analyses, in which the fluorescent-labelled ligand molecules are bound and, subsequently are displaced by the unlabelled competing ligands to measure affinities for both the labelled and unlabelled ligands. The fact that the linear proportion of FP to the percentage bound/free species can be used to determine the IC_50_ (concentration of the unlabelled ligand (or inhibitor) necessary to displace half of the labelled ligand). Subsequently, the corresponding K_i_/K_d_ (K_i_ is the inhibition constant of the unlabelled ligand) can be calculated using the appropriate versions of the Cheng–Prusoff equation [144,145]. K_d_ values measured at different temperatures can be used to estimate binding enthalpy via van’t Hoff analysis [12].

FP technique makes use of single fluorescent label strategy and does not involve the filtration or separation steps and, as thus, requires relatively fewer reagents, smaller amounts of protein, and relatively inexpensive equipment than do SPR and ITC. The other advantages of FP are: the assay protocol is simple (*i.e*., mix-and-read or homogenous), the reaction equilibrium is not disturbed, plates can often be repetitively measured (FP detection does not destroy samples), and the procedure is easily automated. These advantages make this technique well suited to application to HTS of large numbers of unlabelled ligands [12]. However, as a ratiometric method, the response determined by FP is not a direct measure of the binding but rather proportional to the binding [100,146], and this could lead the measured affinity values to be associated with the experimental conditions used [147]. In addition, although the ratiometric measurement makes FP relatively insensitive to the absorptive interferences or inner filter effects, the other effects, e.g., autofluorescence and light scattering, can confound sample FP calculation [139,143,148]. Moreover, the usage of the fluorescent-labelled ligand that may affect the binding behavior, incorrect corrections for non-specific binding, presence of non-binding contaminants or of contaminants that might enhance binding, and the anomalous polarization arising from aggregation-based non-specific binding due to the use of high test compounds concentrations may also impede the actual calculations of binding affinity [12,100,149]. Care must be taken to avoid these potential sources of errors during FP measurement.

### 4.2. Theoretical/Computational Methods

Although experimental techniques can investigate thermodynamic profiles for a ligand–protein complex, the experimental procedures for determination of binding affinity are laborious, time-consuming, and expensive. Modern rational drug design usually involves the HTS of a large compound library comprising hundreds or thousands of compounds to find the lead molecules, but this is still not realistic using experimental methods alone. Most importantly, in the absence of the structural/dynamic data of the protein–ligand complex and the unbound partners, it is difficult to establish a link between the structure and the thermodynamics of the binding event. The contributions of both enthalpic and entropic changes to binding free energy obtained with experiments are the global thermodynamic parameters that reflect the overall heat effect of energy exchange between various species within the system and the redistribution of heat energy upon titration and complex formation. It is therefore necessary to parse the overall heat effect into individual ones such as those of the solvation and desolvation of the protein and ligand, interactions between the binding partners, changes in intramolecular interactions and dynamics, and interactions between the solutes and ions.

Theoretical/computational approaches have enormous potential in providing insights into each of the above effects and in parsing/rationalizing their contributions to the changes in enthalpy, entropy, and free energy. Therefore, it is indispensable to develop and utilize theoretical methods which will not only facilitate the interpretation of the existing experimental data, but also direct the design of new experiments.

In fact, structure-based computational approaches are valuable in all aspects of investigating protein-ligand binding events. For example, if the experimental structure of a protein is unavailable (e.g., GPCR member), theoretical approaches such as homology modelling, threading, or *ab initio* prediction allow for constructing the structural models that can be used for predicting protein-ligand binding. Molecular dynamics (MD) simulations can provide time-dependent changes in atomic coordinates of the protein and ligand in both bound and unbound forms, which are extremely useful in extracting information about the conformational entropy change upon binding. MD simulations allow also for investigations of the non-equilibrium effects that result in the transient conformers, which contribute to the binding event but cannot be readily observed in experiments. Protein-ligand docking methods can quickly predict the most favorable structure of the complex and assess the binding affinity. More accurate prediction of binding affinity can be obtained through free energy calculations, which consider all thermodynamically relevant phenomena such as the protein dynamics/flexibility, explicit inclusion of the solvent, and the difference between protein-ligand interactions in the complex and their interactions with water and counterions in their unbound forms. Next, we will mainly focus on two classes of theoretical methods: the protein-ligand docking and the binding free energy calculations.

#### 4.2.1. Protein–Ligand Docking

Molecular docking is a widely used, relatively fast, and economical computational tool for predicting *in silico* the binding modes and affinities of molecular recognition events [14]. Protein–ligand docking, which is a branch of the molecular docking field, represents a particularly important methodology due to its importance in the current drug discovery process [14,150,151], *i.e.*, virtual screening of large databases of available chemicals in order to select likely drug candidates [152]. Therefore, protein–ligand docking has been an active area of research over the past 20 years, leading to a great variety of available docking software packages. Some well-established ones exist, such as AutoDock [153], GOLD [154], DOCK [155,156], FlexX [157], and Glide [158], which implement different algorithms to solve the docking problem and have a large and rather stable number of users. For an exhaustive review of literature-cited software packages, the reader is referred to [14].

Protein–ligand docking methods contain two essential components: the search algorithm and the scoring function. The former is responsible for searching through different ligand conformations and orientations (poses) within a given target protein; the latter is responsible for estimating the binding affinities of the generated poses, ranking them, and identifying the most favorable binding mode(s) of the ligand to the given target.

##### Search Algorithms and Their Challenges

In theory, the search space for protein–ligand binding should consist of all possible conformations of the protein and the ligand in their unbound forms, all possible orientations and conformations of the ligand within a given protein conformational state, and all possible conformations of the protein paired with all possible conformations of the ligand [159]. However, it is impossible to exhaustively explore the search space with the current computational power and search algorithms. In a search algorithm, two critical elements are the speed and the effectiveness in covering the relevant conformational space [160]. The biggest challenge is how to efficiently deal with the flexibility of the protein. The reasons for this are: (i) there is a large number of degrees of freedom that have to be considered, but neglecting them often leads to poor docking results in terms of binding pose prediction; and (ii) the extensive sampling of the protein conformational space with MD simulations requires a large amount of computational time, which improves the accuracy but lowers the efficiency. Nevertheless, the search algorithms have evolved from the pure rigid-body methods to the flexible-ligand, and further to the flexible ligand–flexible protein methods [14].

The rigid-body algorithms, which are the simplest approach to sampling the conformational space resulting from a ligand–protein association, treat both the ligand and the protein as the rigid body and explore only the six degrees of rotational and translational freedom of the ligand [14]. These approaches are used by ZDOCK [161], MDock [162,163], older versions of DOCK [164], *et al.* Actually, so-called rigid-body algorithms still consider the ligand flexibility by pre-computing ensembles of putative ligand conformations, followed by rigidly docking each conformation to the protein receptor of interest [13,165].

The flexible-ligand algorithms consider only the ligand flexibility but neglect the protein flexibility. In these algorithms, possible ligand conformations may be generated on-the-fly in the binding cavity of the protein [166] (e.g., DOCK [156] uses of this approach), or using the fragmentation-based method in which the multiple rigid fragments located in the binding cavity are rotated and linked [167] (e.g., LUDI [167] uses this approach). In addition, the knowledge-based [168] and force-field energy evaluation [169,170] approaches, and the hierarchical filters [158,169], have also been often used in sampling the ligand conformations.

The flexible ligand–flexible protein algorithms introduce the flexibility of the target protein in addition to that of the ligand and as thus represent the high-end approach. There are several strategies to handle the protein flexibility. One simple approach to emulate receptor flexibility is to dock a ligand to the multiple static structures of the same protein [171], which can be either the experimentally determined (e.g., through X-ray crystallography or NMR) conformations or the conformational ensemble generated by simulation methods (e.g., MD, MC, or normal mode analysis). Of interest is that such a simple strategy is in line with the conformational selection mechanism of protein-ligand binding. Another strategy, which is in line with the induced fit mechanism, is the energy minimization performed using the MC methods or gradient descent minimization [172]. In addition, local/partial conformational flexibility can be accounted for by exploring only the conformational space of the critical residues of the protein [173,174]. For example, through searching the rotamer libraries of amino acid side chains surrounding binding cavity, one may obtain the alternate but energetically reasonable protein conformations [175]; through introducing the “soft core potential” and allowing a certain overlap between protein and ligand, soft docking can detect small-scale rearrangements of the side-chains on the protein [176].

##### Scoring Functions and Their Challenges

Scoring functions are fast, approximate mathematical methods used to assess the binding affinity (generally through measuring the strength of noncovalent interactions) between the protein and the ligand after docking. Of note is that the scoring function itself can be utilized within the search algorithm to accelerate the process of binding mode prediction [156]. A perfect scoring function would be able to predict the binding free energy of the protein–ligand complex and simultaneously should be fast enough to allow its application to the high-throughput virtual screening. However, this goal is challenged by both the computational methods and the computational resources. In order to calculate accurately the binding free energy, many different physical interactions (especially those involving the solvent) as well as the entropic effects should be included, but this is unrealistic due to the algorithm’s complexity and the need for large amounts of computation. As a result, scoring functions incorporate a number of simplifications to reduce the complexity and computational intensity at the cost of accuracy [14].

There is a large number of scoring functions available for protein–ligand docking studies, of which the most commonly used can be divided into three general classes: the force-field-based, the empirical, and the knowledge-based (or statistical potential) scoring functions.

In the force-field-based approaches, physical-based functional forms and parameters (*i.e*., the force fields) derived from experiments and quantum mechanical calculations are employed to estimate the binding affinities [177]. In order to reduce the complexity, usually only the strengths of intermolecular noncovalent interactions (the enthalpic contribution) in the complex are estimated. However, a more accurate estimate of binding affinity should include the changes upon binding in intramolecular interactions of the two binding partners and in the interactions involved in the solvent and, particularly importantly, the entropic effects. The solvent effect can be accounted for either by treating water molecules explicitly or using the implicit (or continuum) solvent models such as Poisson–Boltzmann surface area (PBSA) model [178,179] and the generalized-Born surface area (GBSA) model [180,181].

Compared to the computationally intensive force-field-based approaches, empirical scoring functions provide a higher-speed alternative due to a greater degree of simplification. Empirical scoring functions are based on the parameterization of various types of interactions as favorable or unfavorable energy terms via regression or machine learning methods [13,182]. These energy terms may include van der Waals and electrostatic energies, hydrophobic contacts, hydrophilic contacts, number of hydrogen bonds, number of rotatable bonds that are immobilized upon complex formation, or change in solvent accessible surface area (SASA) upon complex formation. Of note is that these terms do not capture the underlying physics of the interactions but are only the simplified terms attempting to approximate the favorable or unfavorable contributions to the binding affinity. The major challenge of empirical scoring functions is to develop accurate energy terms that are fast enough to allow conformational search. Since empirical scoring functions comprise many energy terms, another challenge is how to avoid double-counting problems (over-fitting) [183]. In addition, their general applicability may also depend on the training set due to the nature of fitting binding affinities to a small dataset [183].

The knowledge-based scoring functions are based on the hypothesis that the close inter-atomic interactions between binding partners that occur more frequently than those expected by a random distribution are likely to be energetically favorable and, as thus, make favorable contributions to the binding affinity [184]. In other words, the statistically observed close contacts in a training set containing suitable samples (obtained from protein structural databases) are used to derive the statistical potentials. Although several problems (*i.e.*, reference state problem, sparse data problem, and other problems arising from the physically non-rigorous assumptions) exist in the implementation of statistical potentials [13], the knowledge-based scoring functions have the advantages of being simpler and faster than force-field-based potentials, being less prone to over-fitting compared to the empirical functions, and performing well in cases where the training set provides poor coverage.

Because each scoring function has its own advantages and disadvantages, and none of them is perfect in terms of the accuracy and general applicability, the consensus scoring strategy has been introduced to improve the probability of finding correct solutions by combining the scores from multiple scoring functions [183,185,186].

#### 4.2.2. Free Energy Calculations

Free energy calculations of the protein-ligand binding try to compute the binding free energies based on the principles of statistical thermodynamics. Such calculations are commonly based on extensive computational simulations (*i.e.*, MD or MC) of the protein and ligand and, as such, require computational efforts several orders of magnitude higher than the traditional scoring functions. As a reward for the highly intensive computation, the results of free energy calculations ought to be reliable and almost quantitative. The main advantages over faster scoring functions are that the free energy calculations include both the energetic (*i.e.*, potential energy and solvation energy) and entropic (*i.e.*, dynamics/flexibility of both protein and ligand, and solvent effects) contributions, and require no case-by-case parameter fitting [3,187]. Anyway, the accurate prediction of binding free energy using the calculation methods, despite being an ambitious goal, would revolutionize their applications in basic research and in drug design and discovery.

Free energy calculations rely on the fundamental relationship between Helmholtz free energy F and the partition function Z [3]:

(11) $$F=-k_{B}\text{TlnZ}$$

where k_B_ and T is Boltzmann’s constant and temperature, respectively. When the system is treated in terms of the classic approximation of statistical thermodynamics, the partition function can be expressed as configurational integral [188]:

(12) $$Z=\frac{1}{h^{\text{3N}}\text{N!}}\iint e^{\frac{-{H(\vec{p}}^{N}{\vec{r}}^{N})}{k_{B}T}}{d\vec{p}}^{N}{d\vec{r}}^{N}=\text{factor}\int e^{\frac{-{V(\vec{r}}^{N})}{k_{B}T}}{d\vec{r}}^{N}$$

where h is Planck’s constant, *N* is the number of atoms or particles in system, and N! is only present for indistinguishable particles. ${\vec{p}}^{N}$ = (${\vec{p}}_{1}$, ${\vec{p}}_{2}$…${\vec{p}}_{N}$) and ${\vec{r}}^{N}$ = (${\vec{r}}_{1}$, ${\vec{r}}_{2}$…${\vec{r}}_{N}$), which represent the conjugate momenta and Cartesian coordinates of all N atoms, respectively. The Hamiltonian ${H(\vec{p}}^{N}{,\vec{r}}^{N})$ consists of the kinetic energy K(${\vec{p}}^{N}$) and the potential energy V(${\vec{r}}^{N}$) of the system. The latter describes the interactions between the various atoms in system (*i.e.*, potential function). The difference in free energy between two states A and B can be expressed as a ratio of their partition functions [189]:

(13) $$\Delta F=-k_{B}\text{Tln}\frac{Z_{B}}{Z_{A}}$$

If the conformational sampling is carried out under constant temperature and pressure conditions (isothermal-isobaric ensemble), the Gibbs free energy can be obtained.

For further detailed theoretical background of free energy calculations, the reader is referred to [3,15,188,190,191]. Here, we will introduce three main types of calculation methods: the alchemical calculation, the path sampling, and the endpoint methods. It should be noted that the efficiency and accuracy of the calculations could be influenced by whether the implicit or the explicit solvent is used, the length of the simulations, and the choice of calculating the absolute or relative free energy of binding.

##### Alchemical Free Energy Calculations

Alchemical free energy calculations employ unphysical (“alchemical”) intermediates to estimate the free energies of various physical processes. In the case of protein–ligand binding, the ligand is alchemically transmuted into either another chemical species or a non-interacting “dummy” molecule via intermediate, nonphysical stages [192,193,194,195]. A simple alchemical calculation involves using thermodynamic cycles to compute the change in free energy when ligand A is changed to ligand B free in solution (Δ*G*_free_(A→B)) and within the ligand-binding site (Δ*G*_bound_(A→B)). The difference between these two free energy changes is equal to the binding free energy difference of the two ligands (Δ*G*_bind_ (B) − Δ*G*_bind_(A)):

Δ*G*_bound_(A→B) − Δ*G*_free_(A→B) = Δ*G*_bind_ (B) − Δ*G*_bind_(A) = ΔΔ*G*_bind_ (14)

In principle, if the ligand A is transmuted into non-interacting dummy particles, it is possible to compute the ligand’s absolute binding free energy [196], but this is problematic since severe convergence problems occur near the end state of the transformation [3]. In many practical applications of this method, a single alchemical transformation is often broken down into several intermediate steps.

Applications of the alchemical strategy are implemented by the free energy perturbation (FEP) [197], the thermodynamic integration (TI) [198], and the Bennet’s acceptance ratio (BAR) [199] methods, each of which has its own advantage in solving problems of conformational sampling and statistical convergence. For case studies using these methods as well as their challenges and improvements made to them, the reader is referred to [192] and references therein. Here, we mainly introduce the basic principles of the FEP and TI methods.

In the FEP method, the free energy difference between states A and B can be evaluated with the Zwanzig equation [197]:

(15) $$\Delta G^{\text{FEP}}=G_{B}-G_{A}=-k_{B}\text{Tln}{\langle e^{-(V_{B}-V_{A})/k_{B}T}\rangle }_{A}=\;+k_{B}\text{Tln}{\langle e^{-(V_{A}-V_{B})/k_{B}T}\rangle }_{B}$$

where V_A_ and V_B_ are potential functions of the states A and B, respectively, and the triangular brackets denote the Boltzmann-weighted ensemble average generated according to the potential function of the corresponding state. Equation (15) indicates that the potential energy differences can be averaged over an ensemble generated using MD or MC simulations that start from either the state A or the state B, thus allowing to estimate the convergence through comparing free energy results between the forward and backward transformations [3]. FEP calculations only converge properly when a small enough difference exists between the two states. In a practical calculation, the FEP is broken down into multiple small steps (or windows) by simulating the transition from A to B via intermediate states and summing all free energy changes for transition in each step as the total free energy change.

In the TI method, the free energy difference between states A and B is calculated by defining a thermodynamic path connecting them (which is composed of the non-physical coordinate, commonly called λ), simulating the transition from A to B along this path, and integrating the Boltzmann-weighted λ-derivative of the mixed potential function over λ. The potential energy function corresponding to a λ value is defined as:

V(λ) = V_A_ + λ(V_B_ − V_A_) (16)

where the λ ranges between 0 and 1, and thus the potential energy function V varies from V_A_ when λ = 0 to V_B_ when λ = 1. The ensemble average of the derivative of the potential energy function with respect to λ at each value can be obtained through MD or MC simulation. Finally, the integral for the ensemble-averaged derivatives is computed to obtain binding free energy:

(17) $$\Delta G^{\text{TI}}={\int_{0}^{1}\langle \frac{\partial V(\lambda )}{\partial \lambda }\rangle }_{\lambda }d\lambda$$

It should be noted that, unlike calculations with the FEP method, there are no forward and backward transformations in the TI calculations.

##### Path Sampling

The above described FEP and TI methods calculate the free energy change along the non-physical or alchemical reaction coordinates (*i.e.*, involving atom type changes). There also exists a set of methods that aim at computing the binding free energy along a physically possible path associated with the conformational changes. The obtained free energy map/profile along one or more reaction coordinates is traditionally called the potential of mean force (PMF). Since even a small potential barrier along the reaction coordinate can trap the conformation and prevents sufficient sampling of the conformational space, enhanced sampling techniques, e.g., the adaptive biasing force algorithm [200], temperature acceleration [201], umbrella sampling [202], metadynamics [203], and replica exchange [204], may be utilized to improve sampling and speed up the convergence of PMF calculations.

There are several different approaches to obtaining the protein-ligand binding free energy with path sampling. One approach is to sample the dissociation path starting from the bound protein-ligand complex. For example, the smooth reaction path generation (SRPG) method starts with generating a very rough dissociation path followed by smoothening the path and further performing TI calculations [205]; the double decoupling methods involve calculating the work of slowly decoupling the ligand from the binding site and then reintroducing the ligand to the bulk solvent [206,207]. On the contrary, a PMF-based path method performs the binding free energy calculations through initially restraining the ligand into the bound state in the bulk solvent, followed by translating the ligand into the binding site and then releasing restraints completely [208]. Also worth noting is the non-equilibrium methods which are based on the Jarzynski relationship [209,210,211,212]. In these methods, instead of the extensive sampling (equilibrium simulations) at any point in a reaction path, many non-equilibrium simulations are performed to pull continuously the system from the starting to the final conformation. It has been shown that these methods are readily parallelized and applied to alchemical calculations [3,187,213].

##### Endpoint Methods

Endpoint methods attempt to compute binding free energy from simulating only the free and bound states of species but do not consider either the physical or the non-physical intermediates. Therefore, endpoint methods can be more efficient than previously described alchemical calculations and path sampling methods.

One such approach is the linear interaction energy (LIE) method, which is based on the assumption that the binding free energy is dependent linearly on the changes in interaction energy of the ligand with its surroundings [214]. Therefore, only two simulations, one for the ligand in solution (free state), and the other for the ligand in complex with the protein (bound state), are required. The binding free energy is then estimated as:

(18) $$\Delta G^{\text{LIE}}=\alpha \left({\langle V_{\text{vdw}}\rangle }_{\text{bound}}-{\langle V_{\text{vdw}}\rangle }_{\text{free}}\right)+\beta \left({\langle V_{\text{elec}}\rangle }_{\text{bound}}-{\langle V_{\text{elec}}\rangle }_{\text{free}}\right)$$

where the angle brackets indicate Boltzmann averages over generated conformational states, the terms within the parentheses represent the differences in van der Waals (V_vdw_) and electrostatic (V_elec_) interaction energies of the ligand with its environments when the bound and free states are compared, and α and β are empirical parameters that account for the changes in the internal energies of the solvent and the protein on the basis of their reorganization energy in response to the ligand [214]. It has been shown that good correlations between the calculations and the experiments can be obtained when α and β are set to 0.18 and 0.33, respectively [215], and when the system-specific values are used [190,216,217]. Because LIE does not take into acount explicitly the changes in the conformational entropy and the internal energy of the ligand, the main reasons for its success may be due to the pure ligand comparison within a single chemical series, the enthalpy–entropy compensation, or the cancellation of the solute entropy decrease by the solvent entropy gain [15]. However, the parameter dependence of LIE may limit the range of its applications and its predictive power and efficiency.

Another two similar endpoint methods, which have often been applied to the protein-ligand binding free energy calculations, are molecular mechanics Poisson-Boltzmann surface area (MM-PBSA) and molecular mechanics generalized born surface area (MM-GBSA) [218,219]. In the MM-PBSA method, the binding free energy is calculated as:

(19) $$\Delta G^{\text{MM-PBSA}}=\Delta G^{\text{vacu}}+(\Delta G_{\text{PL}}^{\text{solv}}-\Delta G_{P}^{\text{solv}}-\Delta G_{L}^{\text{solv}})$$

where Δ*G*^vacu^ represents the vacuum binding free energy, $\Delta G_{\text{PL}}^{\text{solv}}$, $\Delta G_{P}^{\text{solv}}$, and $\Delta G_{L}^{\text{solv}}$ are the solvation free energies of the protein–ligand complex, free protein, and free ligand, respectively. Typically, three independent MD simulations for the complex, free protein and free ligand may be performed with a molecular mechanics force field and an explicit solvent model, with the three generated trajectories as the basis for calculating the above free energy contributions [220]. Alternatively, a single trajectory obtained from only one simulation of the protein–ligand complex may be used as the basis (trajectories of the nominally free ligand and free protein are derived simply by removing the other partner in the complex trajectory, respectively), which accelerates the convergence of the energy average, a problem confronting the multiple-trajectory approach [15,221].

After stripping the explicit solvent molecules from the ensemble(s), Δ*G*^vacu^ is calculated as:

(20) $$\Delta G^{\text{vacu}}=\langle V_{\mathrm{PL}}\rangle -\langle V_{P}\rangle -\langle V_{L}\rangle -T\Delta S_{\text{solute}}$$

where the $\langle V_{\mathrm{PL}}\rangle$, for example, indicates the Boltzmann-averaged potential energy of the protein-ligand complex computed using the molecular mechanics force filed, and the Δ*S*_solute_ is the change in solute entropy upon binding, including the contributions from Δ*S*_conf_ and Δ*S*_r/t_ as shown in Equation (7). These entropy terms can be estimated with standard statistical thermodynamics models, e.g., normal mode [222] or quasi-harmonic analysis [223] for Δ*S*_conf_, and Sackur-Tetrode equation [224] or alternative models [22] for Δ*S*_r/t_.

The solvation free energy terms are computed by the PBSA implicit solvent model, in which the Δ*G*_solv_ is divided into two components:

(21) $$\Delta G_{\text{solv}}=\Delta G_{\text{PB}}^{\text{elec}}+\Delta G_{\text{SASA}}^{\text{hydr}}$$

where $\Delta G_{\text{PB}}^{\text{elec}}$ is the electrostatic contribution to the solvation free energy calculated with the PB method, and $\Delta G_{\text{SASA}}^{\text{hydr}}$ is an empirical term of the hydrophobic contribution that is linearly dependent on the SASA. Alternatively, the Δ*G*_solv_ can be computed by the GBSA implicit solvent model, which is faster and often provides a better accuracy, although its results are sensitive to details in the calculations [225] and, its performance in calculating absolute binding free energy is no better than MM-PBSA [222]. A common feature of these two approaches is that the choice of the solute dielectric constant has a large influence on the calculated results. Consequently, this parameter should be carefully determined according to the characteristics of the protein–ligand binding interfaces [222].

The modular nature of MM-PB/GBSA means that the reliability of the calculated results depends on the fortuitous cancellation of errors that may differ from system to system. Therefore, careful checking and comparison of the results with those from experiments or other computational methods may be required. In addition, the overestimate of the entropy decrease by normal mode analysis, the lack of information about the number and entropy of water molecules in the binding site, and severe convergence problems also deteriorate the reliability and efficiency of these two methods. Nevertheless, MM-PB/GBSA methods have been applied successfully to protein–ligand binding studies and have generated some encouraging results. Because of their intermediate position between the empirical scoring and rigorous alchemical calculation methods in terms of both accuracy and computational intensity, MM-PB/GBSA could be useful for post-processing of the docked structures or be used to rationalize the observed differences [225].

## 5. Conclusions

This paper has reviewed extensively three aspects involving protein–ligand recognition and binding: mechanisms, models, and methods. With respect to physicochemical mechanisms, the decrease in total Gibbs free energy of the protein-ligand-solvent system, which depends on a delicately balanced mechanism of opposite effects involving both the enthalpic and entropic contributions, is the global driving force for the binding reaction. Although the entropy–enthalpy compensation has an adverse effect on a large free energy change, the solvent entropy gain (e.g., due to solute desolvation) and the enthalpy decrease (e.g., due to the favorable interactions between the binding partners) can still overcompensate for the unfavorable contributions from the enthalpy increase (e.g., the energy required for desolvation) and the entropy decrease (*i.e*., conformational entropy decrease and the loss of the rotational and translational entropy of the ligand), thus lowering the system free energy and resulting in a funnel-like binding FEL (binding funnel) similar to the protein folding funnel [24,26,29].

With respect to the binding models, the entropy-dominated lock-and-key model, although based on a unrealistic, rigid hypothesis, may solve the question of why a given ligand will bind specifically to a protein out of the many different proteins in the cell [60]. The enthalpy-dominated induced fit model is relatively realistic since it takes into account the protein conformational flexibility, especially the conformational changes surrounding the binding site; however, it may not explain well binding where proteins undergo large conformational changes [60]. The conformational selection model may be a more realistic one because it takes into account not only the inherent protein flexibility, but also the population shift and redistribution of the conformational states/substates. In this model, the selective binding of the ligand to a conformer of the protein resembles the entropy-dominated lock-and-key process, and the following conformational adjustment is essentially the induced fit process dominated by favorable negative enthalpy change. The conformational selection model could be used to interpret the phenomena that a protein can interact with multiple structurally dissimilar but functionally important partners. Of interest is that, for a flexible protein that presents multiple conformational states/substates, either the conformational selection or the induced fit may dominate the ligand binding process, depending on the ligand concentration and the protein conformational transition rate.

With respect to the experimental methods used to investigate the protein-ligand binding, ITC is a gold standard in estimating the binding driving forces and the stability of the protein-ligand complex because of its ability to provide a complete thermodynamic signature/profile of the system studied. SPR can determine directly the kinetic rate constants (k_on_ and k_off_). FP can estimate the equilibrium dissociation constant K_d_ through competition binding analyses. As a result, the latter two experimental methods are used frequently to measure the binding affinity and can even estimate the binding enthalpy if measurements are performed at different temperatures. Development and utilization of the theoretical/computational methods are significant and indispensable because they may, to a large extent, be relatively less laborious, more economic, and faster than experimental methods and, further, can facilitate the interpretation of the existing experimental data and direct the design of new experiments. The protein-ligand docking methods, although less accurate in estimating the binding free energy than the free energy calculation approaches, can quickly and cheaply predict the correct bound conformations and, hence, are particularly suitable for application in high-throughput virtual drug screening. The main challenges confronting docking and scoring include: how to develop a fast and accurate model for the energetics of protein-ligand interactions; how to efficiently account for protein flexibility; how to handle the presence of water molecules [13,14]; and how to treat entropy. Since free energy calculations are based on the principles of statistical thermodynamics and depend on extensive MD/MC simulations, one would hope to obtain higher accuracy with free energy calculations compared to scoring functions within the docking methods. The characteristics of low-efficiency and potential high-accuracy of free energy calculations make them suitable to be applied to more detailed study of protein-ligand interactions. Among the three types of free energy calculation methods, the traditional alchemical calculations are the most accurate and robust but are less efficient methods, while the newer endpoint methods are the fastest but are less accurate methods. The path sampling methods comprise a very promising approach, for which the problems of how to obtain the right path and how to accelerate sampling convergence should be addressed. The common challenges of free energy calculations are to improve the speed of the methods and to increase the accuracy and reliability of calculated results. The development and/or utilization of more sophisticated/accurate force fields (e.g., incorporation of polarization effects in current force fields) [15], more efficient sampling techniques, and more accurate solvent models, and the combining of existing methods to make use of the strong points of individual methods [187], could be the main directions to head in.
